# The preference of women living with HIV for the HPV self-sampling of urine at a rural HIV clinic in Uganda

**DOI:** 10.4102/sajid.v37i1.414

**Published:** 2022-12-02

**Authors:** Agnes Nyabigambo, Roy W. Mayega, Hilbert Mendoza, Aslam Shiraz, John Doorbar, Lynn Atuyambe, Themba G. Ginindza

**Affiliations:** 1 Discipline of Public Health Medicine, School of Nursing and Public Health, University of KwaZulu-Natal, Durban, South Africa; 2 Community Health and Behavioural Sciences, School of Public Health, Makerere University, Kampala, Uganda; 3 Health Economics and HIV/AIDS Division (HEARD), University of KwaZulu-Natal, Durban, South Africa; 4 Department of Epidemiology and Biostatistics, School of Public Health, Makerere University, Kampala, Uganda; 5 Social Epidemiology and Health Policy, Department of Family Medicine and Population Health, University of Antwerp, Antwerp, Belgium; 6 Department of Pathology, University of Cambridge, Cambridge, United Kingdom; 7 Department of Community Health and Behavioural Sciences, School of Public Health, Makerere University, Kampala, Uganda; 8 Cancer and Infectious Diseases Epidemiology Research Unit (CIDERU), College of Health Sciences, University of KwaZulu-Natal, Durban, South Africa

**Keywords:** HPV, HIV, women, ART, VIA

## Abstract

**Background:**

Women living with HIV have a double risk of acquiring cervical cancer (CC) due to repeated human papilloma virus (HPV) infections resulting from reduced immunity, with CC screening being low at 46.7%.

**Objectives:**

To determine the factors associated with the preference for HPV self-sampling using urine as well as establish its feasibility among women living with HIV attending a rural HIV clinic in Uganda.

**Method:**

A cross-sectional study design using quantitative data collection methods was used at the HIV clinic, Luweero District Hospital, among 426 women aged between 30 and 65 years. Data were analysed using descriptive statistics and modified Poisson regression. Urine samples were analysed using a Liferiver high-risk HPV genotyping real-time polymerase chain reaction (PCR) kit to determine the prevalence of the 15 HPV subtypes. Cervical intraepithelial neoplasia 2 (CIN2) was determined by visual inspection under acetic acid (VIA) using the nurse-led approach.

**Results:**

Most women (296/426, 70%) preferred nurse-led screening. Preference for HPV self-sampling using urine was associated with older age (46–65 years) (adjusted prevalence risk ratios [aPRR] 1.59; 95% confidence interval [CI]: 1.13–2.24), history of sexually transmitted infections (aPRR 0.74: 95% CI: 0.55–0.98) and acquisition of CC information from the television (aPRR 1.48: 95% CI: 1.09–2.02). Approximately 97% (68/70) of women living with HIV tested HPV positive with one or more subtypes. The most prevalent subtype of HPV was HPV 58 (87.1%). Only one woman tested positive with VIA.

**Conclusion:**

Nurse-led CC screening is preferred among women living with HIV, and HPV self-sampling using urine is feasible at the HIV clinic. Therefore, educational programmes to reassure the masses about urine HPV self-sampling need to be designed.

**Contribution:**

This study’s findings provide early insights into the merits and demerits of the current HPV sample collection approaches. Hence, HPV testing should be tailored to routine HIV care in rural communities.

## Introduction

Cervical cancer (CC) is the most common cancer among Ugandan women, with the highest age-standardised incidence rate of 54.8 per 100 000 women per year.^1^ Consequently, around 6413 women are diagnosed with CC, and 4301 die annually.^1^ The main causative agents of CC are the high-risk subtypes of human papilloma virus (HPV) such as HPV 16 and HPV 18.^2^ It is estimated that 3.6% of the Ugandan woman population harbours HPV 16 or 18 at any given point in time.^1^ Women living with HIV are more susceptible to persistent HPV infections because of their reduced immunity. Hence, they have a higher risk of developing CC compared to HIV-negative women.^3,4^

Despite this high burden, CC is a preventable form of cancer. The World Health Organization (WHO) recommends screening as one of the secondary prevention measures. Consequently, the Ugandan guidelines recommend annual screening for HPV negative women living with HIV aged from 25 to 49 years.^5^ The three WHO-recommended CC screening modalities include the Papanicolaou test (Pap smear), visual inspection of the cervix with acetic acid (VIA) and HPV testing. Visual inspection of the cervix with acetic acid and HPV testing are the preferred CC screening methods over the Pap smear because of their cost-effectiveness for a low-income country like Uganda.^6^ In July 2021, Uganda adopted the WHO CC screening guidelines, where the HPV test is recommended as a screening test that should be conducted annually in HPV negative women living with HIV.^7^ In Uganda, VIA is still the most common CC screening method for cervical intraepithelial neoplasia (CIN) grading,^8^ which is now a triage test by the new WHO CC screening guideline.^7^ However, VIA effective uptake is hindered by challenges such as inadequately trained health personnel, embarrassment, fear of screening procedures and rural residence.^9^

However, self-sampling for HPV testing has the potential to overcome these barriers and increase the CC screening coverage.^10^ With HPV self-sampling, women themselves collect the sample, either a vaginal swab or urine, which is then tested in the laboratory for high-risk HPV fields.^11^ Because sample collection is done by the woman, challenges related to embarrassment and rural residence are minimised.^10^ Furthermore, there is no need for pelvic examination (VIA); thus, there is no need for a trained health worker, who may be inadequate to conduct CC screening for women living with HIV in Uganda.^12^ Nonetheless, in this study, the nurse-led screening approach is the screening for CIN2 using VIA.^8^ Self-sampling for HPV testing is still a new CC screening method in Uganda, and little is known about the preference and feasibility of the method in Uganda.

Studies conducted in a Ugandan urban clinic setting showed that HPV self-sampling using a vaginal swab had the potential to increase uptake as compared to VIA.^13,14^ However, new literature suggests that urine sampling is potentially a more favourable and reliable method than HPV self-sampling using a vaginal swab, as it is less invasive and thus may increase CC screening coverage even more.^15,16,17,18,19^ The urine HPV self-sampling approach works well within the clinic, with a good storage facility of −21 °C to maintain the viability and quality of the DNA sample.^15,16,17,18,19^ However, no study has been done in Uganda to assess the preference for HPV self-sampling using urine in a rural setting. This study was thus designed to determine the preference and feasibility of high-risk HPV self-sampling using urine among women attending a rural HIV clinic in Uganda.

## Methods

### Study design

A cross-sectional design was used to collect data at one point in time at the HIV clinic at Luweero District Hospital, Luweero district.

### Study site

Luweero District Hospital is located in the Luweero District. The hospital serves the population of greater Luweero, with an HIV prevalence rate of 10.3%. During the time of the study, the clinic served nearly 7000 people living with HIV and/or AIDS. Of these 2557 were female patients above 30 years on antiretroviral therapy (ART). Luweero district is located in the central region of Uganda, where 19% of women have multiple sexual partners, a key risk factor for HIV and HPV transmission.

### Study population

Women were enrolled who were aged between 30 and 65 years of age, had never been screened or not screened within the last three years (those who had screened for > 3 years with normal results) or had abnormal screening results, attended the HIV clinic and consented to participate in the study. The study excluded all women who were pregnant, had a prior or current diagnosis of CC, were in their menstrual periods or had a hysterectomy, had CC screening within the last three years or declined consent. Approximately 750 women had never been screened, had not been screened within the last three years, had abnormal screening results or had been screened for more than three years with normal results.

### Sample size

Using the Kish Leslie’s formula,

$$n=z^{2}\text{pq}/d^{2}$$ [Eqn 1]

where *z* = *Z* score corresponding to 5% level of significance (1.96); *p* = the proportion that prefer HPV self-sampling of urine in an HIV clinic (46.7% or 9%); *d* = 5% precision (0.05); *q* = 1−*p* = (1−0.467) = 0.533. The sample size was calculated to be 383 women. Including a 10% nonresponse rate, the sample was estimated to be 426.

In order to answer the question of feasibility, a random sample of 70 women who preferred HPV self-sampling of urine in an HIV clinic was selected for urine sample collection and HPV laboratory analysis.

### Sampling and data collection procedures

#### Sampling procedures

An electronic sampling frame of women aged between 30 and 65 years was obtained from the Luweero Health Centre IV (HC IV) HIV clinic. Women’s history of CC screening was reviewed in their files. All women who met the inclusion criteria were included in the sampling frame. Because the study was carried out at the hospital, systematic sampling was used. Using patient identification (ID) numbers and contact information, a list of phone numbers was obtained. Random digit IDs with phone numbers were generated in Excel (Microsoft Corporation, Redmond, Washington, United States). The first study participant was selected using simple random sampling with the ID and phone number. The subsequent participants were systematically selected using a sampling interval, *k* = 750/426 = 2. Therefore, every second ID number on the list after the selection of the first ID number was included in the sample until the required number of respondents was attained. Upon obtaining written informed consent, the midwife educated the woman about the urine self-sampling approach and the VIA and Pap smear provider-led screening approaches. A semistructured questionnaire was used to collect data from all women who were systematically sampled.

A systematic random sampling was used to select 70 women living with HIV to be tested for pregnancy among women who had preferred the urine self-sampling approach. However, the first woman to participate was randomly selected, and the subsequent participants were systematically selected using a sampling interval of 2. Every selected woman was contacted, and informed consent for both urine HPV self-sampling and VIA was obtained by the midwife. The women who accepted to be screened for both urine HPV and VIA collected early void urine which was subjected to pregnancy test using human chorionic gonadotropin (HCG). Women who were HCG negative were subjected to VIA, which was conducted by the nurse. Because the WHO guideline does not recommend conducting VIA on pregnant women, this study excluded women who had an HCG positive result.

The samples of urine samples from women with an HCG-negative result were stored and shipped to a central laboratory at Makerere University, Department of Microbiology (Integrated Biorepository of H3 Africa) and later shipped to the University of Cambridge, Department of Pathology, at 2 °C – 8 °C. All women who were both HPV- and VIA-positive were referred to the Uganda Cancer Institute for Pap smear and further management. The HPV testing focused mainly on the 15 known high-risk subtypes, and these included HPV 16, 18, 16, 18, 58, 52, 66, 68, 51, 45, 73, 35, 59, 31, 53, 33 and 39.

#### Laboratory procedures

All urine samples were self-collected by women living with HIV at Luweero HC IV. The samples were stored at −21 °C at the regional lab hub at Luweero HC IV. These samples were shipped to Makerere University Department of Microbiology (Integrated Biorepository of H3 Africa) in a cold chain for initial concentration and storage. The concentrated urine was shipped to the University of Cambridge, Department of Pathology, and was stored at 4 °C before analysis. The urine at the University of Cambridge was further reconcentrated to 200 μL. Using the Qiagen amp^R^ DNA mini kit, HPV DNA was extracted from the concentrated urine. The Liferiver high-risk HPV genotyping real-time polymerase chain reaction (PCR) kit (TD-0324-04) was used for HPV DNA, typing, and sequencing. This kit tests all the 15 known high-risk subtypes (HPV 16, 18, 16, 18, 58, 52, 66, 68, 51, 45, 73, 35, 59, 31, 53, 33 and 39).

**The concentration of the urine, lysis of human papilloma virus cells and human papilloma virus DNA extraction:** The centrifuge was always cooled to 4 °C before starting the experiment. The fast-temperature mode was used, and the centrifuge was at 4 °C to 3900 revolutions per minute (rpm) for 10 min. To concentrate the urine, each sample of urine was transferred into the Amicon Ultra-15 centrifugal filter unit. The tubes were placed and balanced well in the centrifuge set at 4 °C, 3900 rpm, and spun for 10 min until the volume is 200 μL. An IVYX Scientific pipette of 100 μL – 1000 μL was used to transfer the 200 μL to a 1.5 Eppendorf tube. To the concentrated urine solution, 200 μL of lysis buffer plus 20 μL of proteinase K was added to lyse the HPV cell to release the DNA. This process was conducted in the virus room under the laboratory biosafety cabinet.

Using the Qiagen amp^R^ DNA mini kit, 400 μL of 100% ethanol was added to the solution. This mixture was applied to Q1 Amp column-centrifuge at 6000 rpm for 1 min and the entire volume of the mixture was run through the column. The collection tube was replaced and 500 μL of AW1 was added and centrifuged at 600 rpm for 1 min. The mixture was transferred to a new collection tube and centrifuged at 20 000 rpm for 1 min. The columns were placed in 1.5 mL Eppendorf tubes, then 50 μL of butter AE was added and incubated for 1 min at room temperature; it was then centrifuged at 6000 rpm for 1 min, and the DNA was stored at 21°C.

**Liferiver human papilloma virus typing protocol:** The Liferiver kit was placed at room temperature for 15 min. Eppendorf tubes were labelled for each sample using the patient number as follows + 16, 18, 39, 45. For example [23, 16; 23, 18; 23, 39; 23, 45] and sterile water (H**2**O) in Eppendorf tubes was used as the control, labelled W, 16; W, 18; W, 39; W, 45. Each reagent was gently mixed and centrifuged for 5 s. To avoid contamination, the control sample was dealt with first. The master-mix Eppendorf tubes label (n16; n18; n39; n45) was prepared. Sterile water (H**2**O) in Eppendorf tubes was used as the control labelled. The master mix was added to each labelled tube, gently mixed and centrifuged for 5 s. Ten microliters of the master mix were added to the labelled control first and then to the labelled sample tubes. The extracted DNA was added in the control and sample labelled tubes; the master mix and DNA added together were gently mixed and centrifuged for 5 s. The DNA was then put on the amplification plate, covered and pressed completely.

**Amplification and sequencing of DNA on the plate:** The centrifuge was cooled first to 4 °C and the amplification plate with the master mix and DNA together was centrifuged to 1500 rpm for 5 min at 4 °C. To avoid contamination in the laboratory, especially during typing, the control sample was dealt with first. The PCR-Go to Quantistudio machine was programmed to sequence the master mix and the DNA. The analysis of the 15 hr-HPV subtypes was then established.

### Follow-up procedures

Only patients who tested HPV positive with suspicious precancerous condition were referred to the Uganda Cancer Institute for Pap smear to further grade the suspicious precancer. These patients were followed up and their results were obtained.

### Variables

The main outcome variable was the preference for HPV self-sampling. Upon educating women on the HPV self-sampling, VIA Nurse-led screening approach and their benefits, one question was asked on which of the two approaches they would prefer. The secondary outcome was the HPV test result (positive or negative). The independent variables included individual factors like age, parity, level of education, CD4 counts, HCG results, viral load, ART status, sexually transmitted infection (STI) status, ART adherence status, fear of finding disease, type of HPV 16/18. In addition, health system factors that were considered included distance to the health facility, appointment schedule, trained health workers, referral, and return of laboratory tests. Lastly, perceived embarrassment of the screening procedure were included as a community factor.

### Data management and analysis

Data were entered in Research Electronic Data Capture (REDCap; Vanderbilt University, Nashville, Tennessee, United States), verified and cleaned daily as it was uploaded from the field. Descriptive statistics were used to analyse the outcome variables. A chi-square test was used to ascertain the associations at the bivariable level of analysis. Since the level of HPV self-sampling preference was 30.5% (130/426), modified Poisson regression was the most appropriate approach to determine independent variables significantly associated with preference for CC screening. Using logistic regression would have overestimated the odds ratios and hence a high negative or positive effect. Independent variables with *p*-values less than 0.2 at bivariate chi-square analysis qualified to be included in the regression model. Tests for collinearity were performed and variables with Pearson *r* < 0.4 were included in the final modified Poisson regression model to ensure the precision of estimates, and measures of association were adjusted prevalence risk ratios (aPRR) at a 95% confidence interval (CI). The model was built using a step-wise procedure for selecting independent variables and by adding and removing variables from the model until the model with the lowest Akaike’s information criterion (AIC) was obtained. All statistical analyses were performed using Stata 14 (StataCorp LLC, College Station, Texas, United States).

Descriptive statistics were used to ascertain the feasibility of HPV self-sampling using urine. The results have been presented as frequencies, percentages and graphs to describe the HPV self-sampling approach.

### Quality control

The University of Cambridge worked closely with researchers at Makerere University School of Public Health in Uganda to develop the protocol, procedures and tools. All study staff were trained on research ethics and procedures for reviewing HIV records to identify potentially eligible clients, screening potential participants and establishing eligibility and principles of interviewing; they were given practice using REDCap for data collection and an orientation to policies, procedures and logistics for the study.

### Ethical considerations

The study was approved by Makerere University, School of Public Health Higher Degrees Research and Ethics Committee (reference number 599), and the Uganda National Council of Science and Technology (reference number HS2515). The study team had been trained in the protection of human subjects. Therefore, research ethical principles were maintained during the implementation of the study. All participants provided informed consent and were allowed to withdraw consent at any time. Study numbers were used to delink information from the recruited study participants.

## Results

### Preference for human papilloma virus sample collection procedure

**FIGURE 1:**
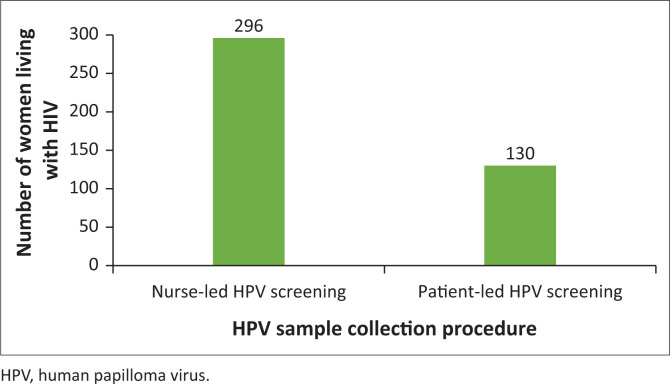
Preference for human papilloma virus sample collection procedure among women living with HIV at a rural HIV clinic.

The majority (69.5%) of the women preferred to be screened by a nurse, while 30.5% (130/426) preferred HPV self-sampling using urine (Figure 1).

### Individual factors associated with preference for human papilloma virus self-sampling using urine

Results in Table 1 show that 72.1% of the participants were within the reproductive age (30–45 years), 99.1% had never accessed any CC screening services, 72.1% had at least had a live birth, 54.5% had CD4 cell counts below 500 cells/mm^3^, 93.7% had an undetectable viral load and 84.7% were not alcoholic. Chi-square analysis of each predictor factor and the preference for HPV self-sampling using urine showed that age, CD4 cell count, history of STI and increased vaginal discharge were significantly associated with the preference for HPV self-sampling using urine.

**TABLE 1:** Individual factors associated with preference for human papilloma virus sample collection procedure.

Individual factors	Total	Preference for HPV screening				Chi-square test (*df*) person *χ*^2^	*p*
		Nurse-led		Patient-led			
		*n*	%	*n*	%		
**Age**							0.029*
30–45 years	324	234	72.2	90	27.8	-	-
46–65 years	102	62	61.0	40	39.0	4.79	-
**Marital status**							0.824
Not married	262	181	69.1	81	30.9	-	-
Married or living with a partner	164	115	70.1	49	29.9	0.051	-
**Level of education**							0.746
Primary education	307	215	70.0	92	30.0	-	-
Secondary education	92	64	70.0	28	30.0	-	-
Tertiary education	27	17	63.0	10	37.0	0.585	-
**Currently on ART**							0.077
No	7	7	100.0	0	0.0	-	-
Yes	419	289	69.0	130	31.0	3.126	-
**Accessed CC screening services**							0.183
No	422	292	69.2	130	30.8	-	-
Yes	004	4	100.0	0	-	1.773	-
**CD4 cell count (cells/mm^3^)**							0.006**
< 500	128	98	76.6	30	23.4	-	-
> 500	107	64	59.8	43	40.2	7.635	-
**Detectable viral load**							0.162
Yes	27	22	81.5	5	18.5	-	-
No	399	274	68.7	125	31.3	1.957	-
**History of any STI**							0.035*
No	213	138	64.8	75	35.2	-	-
Yes	213	158	74.2	55	25.8	4.428	-
**Smoker**							0.554
No	413	286	69.3	127	30.7	-	-
Yes	13	10	76.9	3	23.1	0.350	-
**Drinks alcohol**							0.807
No	361	250	69.3	111	30.7	-	-
Yes	65	46	70.8	19	29.2	0.060	-
**Number of live births (parity)**							0.872
≤ 5 children	307	214	69.7	93	30.3	-	-
> 5 children	119	82	68.9	37	31.1	0.0258	-
**Gravity**							0.986
≤ 6 pregnancies	275	191	69.6	84	30.6	-	-
> 6 pregnancies	151	105	69.5	46	30.5	0.0003	-
**Increased vaginal discharge**							0.026*
No	317	211	66.6	106	33.4	-	-
Yes	109	85	78.0	24	22.0	4.989	-
**Disclosed HIV status**							0.234
No	29	23	79.3	6	20.7	-	-
Yes	397	273	68.8	124	31.2	1.417	-

Note: *P*-values in bold are statistically significant with a *p*-value less than 5%. Age - mean ± s.d. = 40.78 ± 8.23; CD4 cell count (cells/mm^3^) - mean ± s.d. = 547.9 ± 987.5; number of live births (parity) - mean ± s.d. = 4.3 ± 2.3; gravity - mean ± s.d. = 5.7 ± 2.9.

*n*, total number of participants; s.d., standard deviation; *n*, frequency; χ^2^, chi-square; HPV, human papilloma virus; CC, cervical cancer; ART, antiretroviral therapy; STI, sexually transmitted infection; *df,* degree of freedom.

* , *p* < 0.05;

** , *p* < 0.0001.

### Sources of information associated with preference for human papilloma virus self-sampling using urine

The source of information associated with preference for HPV self-sampling using urine was receiving information on CC screening from television and community health workers (CHWs) (see Table 2).

**TABLE 2:** Chi-square analysis of sources of information on cervical cancer associated with preference for human papilloma virus sample collection procedure among women living with HIV at a rural HIV clinic.

Sources of information on CC	Total	Preference for HPV screening				Chi-square test (*df*) person *χ*^2^	*p*
		Nurse-led		Patient-led			
		*n*	%	*n*	%		
**A health worker at the HIV clinic**							0.201
No	45	35	77.8	10	22.2	-	-
Yes	381	261	68.5	120	31.5	1.6324	-
**Family or friends**							0.486
No	346	243	70.2	103	29.8	-	-
Yes	80	53	66.3	27	33.7	0.486	-
**Radio**							0.368
No	199	134	67.3	65	32.7	-	-
Yes	227	162	71.4	65	28.6	0.812	-
**Television**							0.054*
No	346	247	71.4	99	28.6	-	-
Yes	78	47	60.3	31	39.7	3.709	-
**Community health worker**							0.009**
No	279	182	65.2	97	34.8	-	-
Yes	147	114	77.6	33	22.4	6.889	-

Note: *P*-values in bold are statistically significant with a *p*-value less than 5%.

*n*, frequency; *χ*^2^, chi-square; HPV, human papilloma virus; CC, cervical cancer; *df*, degree of freedom.

* , *p* < 0.05;

** , *p* < 0.0001.

### Risky sexual factors associated with preference for human papilloma virus self-sampling using urine

The number of sexual partners was significantly associated with the preference of HPV self-sampling using urine among women living with HIV at a rural HIV clinic (Table 3).

**TABLE 3:** Chi-square analysis of risky sexual factors associated with preference for human papilloma virus sample collection procedure among women living with HIV at a rural HIV clinic.

Risky sexual factors	Total	Preference for HPV screening				Chi-square test (*df*) person *χ*^2^	*p*
		Nurse-led		Patient-led			
		*n*	%	*n*	%		
**Type marital union**							0.738
Monogamy	94	65	69.2	29	30.8	-	-
Polygamy	132	94	71.2	38	28.8	0.1121	-
**Had sexual intercourse within the previous 12 months**							0.249
No	138	91	65.9	47	34.1	-	-
Yes	287	205	71.4	82	28.6	1.327	-
**Number of sexual partners for a lifetime**							0.557
≤ 5 partners	332	233	70.2	99	29.8	-	-
> 5 partners	94	63	67.0	31	33.0	0.3449	-
**Partner’s first sexual encounter**							0.010*
No	273	178	65.2	95	34.8	-	-
Yes	153	118	77.1	35	22.9	6.573	-

Note: *P*-values in bold are statistically significant with a *p*-value less than 5%.

*n*, frequency; *χ*^2^, chi-square; HPV, human papilloma virus; *df*, degree of freedom.

* , *p* < 0.01.

### Multivariate analysis of factors associated with preference for human papilloma virus self-sampling using urine

After controlling for all other factors (Table 4), four factors were found to be significantly associated with the preference of urine high-risk HPV self-collection compared to a nurse-led approach. These include age, history of having an STI, ever hearing about CC screening on television and from a village health team (VHT). Women aged 46–65 years were 1.6 times more likely to prefer HPV self-sampling using urine compared to women aged 30–45 years (PRR = 1.59; 95% CI: 1.13–2.24, *p* = < 0.0001). Those with a history of having an STI in the previous 12 months were 26% less likely to prefer HPV self-sampling using urine compared to women with no history of STIs (PRR = 0.74; 95% CI: 0.55–0.98, *p* = < 0.05). Furthermore, those who had heard about CC screening on television were 1.48 times more likely to prefer HPV self-sampling using urine compared to their counterparts. On the other hand, women sensitised by the CHW on CC screening were 39% less likely to prefer HPV self-sampling using urine compared to those who were not sensitised by the CHW.

**TABLE 4:** Prevalence risk ratios of factors associated with preference for human papilloma virus sample collection procedure among women living with HIV at a rural HIV clinic.

Variable	Total	Preference for HPV screening				Unadjusted PRR	Adjusted PRR
		Nurse-led		Patient-led			
		*n*	%	*n*	%		
**Age**							
30–45 years	324	234	72.2	90	27.8	1	1
46–65 years	102	62	61.0	40	39.0	0.66 (0.47–0.92)*	1.59 (1.13–2.24)***
**Marital status**							
Not married	262	181	69.1	81	30.9	1	1
Married or living with a partner	164	115	70.1	49	29.9	1.0 (0.72–1.30)	1.07 (0.79–1.43)
**Level of education**							
Primary education	307	215	70.0	92	30.0	1	1
Secondary education	92	64	70.0	28	30.0	1.02 (0.71–1.45)	1.03 (0.70–1.51)
Tertiary education	27	17	63.0	10	37.0	1.57 (0.96–2.56)	1.57 (0.96–2.56)
**History of any STI**							
No	213	138	64.8	75	35.2	1	1
Yes	213	158	74.2	55	25.8	0.73 (0.54–0.98)*	0.74 (0.55–0.98)*
**Number of live births (parity)**							
≤ 5 children	307	214	69.7	93	30.3	1	1
> 5 children	119	82	68.9	37	31.1	1.03 (0.75–1.45)	0.99 (0.62–1.57)
**Gravity**							
≤ 6 pregnancies	275	191	69.6	84	30.6	1	1
> 6 pregnancies	151	105	69.5	46	30.5	1 (0.74–1.35)	0.87 (0.56–1.24)
**Heard about CC on television**							
No	346	247	71.4	99	28.6	1	1
Yes	78	47	60.3	31	39.7	1.39 (1.01–1.91)*	1.48 (1.09–2.02)**
**Heard about CC from VHT**							
No	279	182	65.2	97	34.8	1	1
Yes	147	114	77.6	33	22.4	0.64 (0.46–0.91)*	0.61 (0.42–0.88)***

Note: *P*-values in bold are statistically significant with a *p*-value less than 5%. AIC: 1.28; Log pseudolikelihood: −260.54.

*N*, frequency; HPV, human papilloma virus; PRR, prevalence risk ratios; CC, cervical cancer; VHT, village health team; STI, sexually transmitted infection.

* , *p* < 0.05;

** , *p* < 0.01;

*** , *p* < 0.0001.

### Feasibility of the human papilloma virus self-sampling using urine

#### The ability of women living with HIV to conduct human papilloma virus self-sampling of urine

All 70 women were able to collect 50 mLs of early void urine that was stored and shipped to the laboratory for analysis for the 15 hr-HPV subtypes.

#### Prevalence of human papilloma virus and cervical lesions among women living with HIV

**FIGURE 2:**
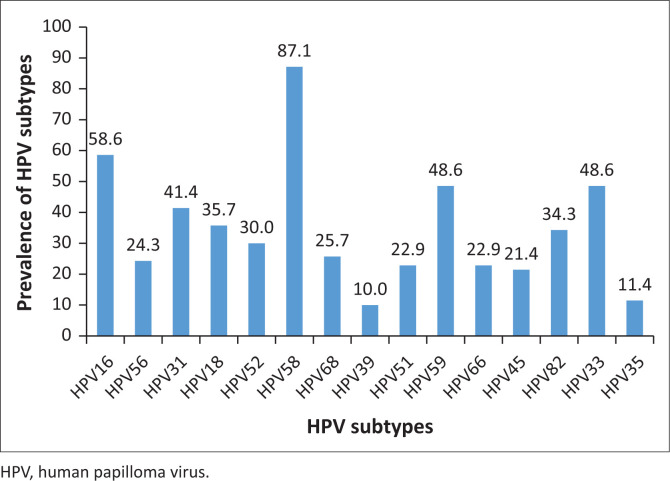
The prevalence of human papilloma virus subtypes among women living with HIV at a rural HIV clinic.

The majority of women (97%) tested positive for HPV with one or more subtypes. The most prevalent subtype was HPV 58 (87.1%), followed by HPV 16 (58.6%), HPV 59 (48.6%) and HPV 33 (48.6%) (Figure 2). However, only one woman tested positive with VIA and the woman was diagnosed with CIN2 using Pap smear. Human papilloma virus screening using urine is feasible at an HIV clinic.

## Discussion

This study aimed to determine the preference and feasibility of high-risk HPV self-sampling of urine among women attending a rural HIV clinic in Uganda. It revealed that the majority of the women preferred nurse-led HPV screening to HPV self-sampling using urine. Factors associated with the preference of HPV self-sampling using urine to nurse-led CC screening were older age (46–65 years), history of having an STI, acquisition of CC information from a CHW and acquisition of CC information from the television.

The study showed that the majority of women living with HIV preferred a nurse-led approach of VIA screening over HPV sampling using urine (69.5% vs 30.5%). The nurse-led screening approach of VIA was preferred, possibly because women anticipated immediate action for treatment in case CIN2 was identified by the nurse. Similar studies in Botswana (81% vs 19%) and Cameroon (62% vs 29%) also showed the same results.^20,21^ Trust in health personnel could be an indirect indication of the lack of confidence about the accuracy of the self HPV urine test. Therefore, there is a need for more educational initiatives targeted to reassure women about the accuracy of the self HPV urine test to increase their confidence.

Older age was significantly associated with a preference for HPV self-sampling using urine as compared to the nurse-led screening. It is more likely that the older women have previously undergone nurse-led CC screening as compared to their younger counterparts. This could be the reason why HPV self-sampling using urine may be preferred as a future screening test as compared to a first-time screening test among rural women living with HIV. Similar studies in the Netherlands^22^ have also shown that HPV self-sampling may be more preferred as a future screening method.

Women who received information on CC from a CHW were less likely to prefer HPV self-sampling using urine. Women living with HIV are educated about early diagnosis and treatment of any disease. They could have perceived that being screened by a nurse using VIA would provide an instant diagnosis and plan for treatment compared to the HPV test, whose results have a longer turnaround time. This could be because CHWs emphasise the importance of health worker–oriented services, such as the nurse-led VIA screening, during their health education sessions with the community. Furthermore, HPV self-sampling using urine is still a novel approach in Uganda; thus, CHWs may not know the screening method. This, however, demonstrates the potential role CHWs can play in educating women about HPV urine sampling to increase preference for HPV self-sampling using urine. Similar studies done in rural settings showed that CHWs played a critical role in increasing the acceptance of HPV self-sampling methods.^23,24^ Also, this study revealed that participants who heard information on CC from television were likely to prefer HPV self-sampling using urine. This could be because television is an appropriate communication channel to visually demonstrate how HPV self-sampling using urine can be done, thus increasing their confidence in the screening method.

It was feasible to conduct HPV self-sampling of urine in a rural HIV clinic. This was evidenced by the ability of women to collect early void urine. Additionally, the availability of laboratory space for storage before shipment to the a laboratory maintained the state of the samples before analysis. The prevalence of hr-HPV and hr-HPV multiple infections was extremely high among women living with HIV.^25^ The findings are similar to the study in South Africa’s Eastern Cape province, where HIV-positive women have a significantly higher hr-HPV prevalence (40.6%, 63/155).^26^ These findings are concurrent with previous studies that have shown an extremely high prevalence of hr-HPV and hr-HPV multiple infections, ranging from 37% – 100% to 42% – 75%.^25^ These study’s findings were slightly similar to the findings in a study conducted in Mali, with a high prevalence of hr-HPV infection (63%).^25^ Additionally, the same study shows an unusual distribution, with HPV 31, HPV 56 and HPV 52 being the most common after HPV 33. The HPV distribution in the study of Mali is different from the current study among HIV-infected women with high prevalences of HPV 58, HPV 16, HPV 59 and HPV 33.^27^ Therefore, clinic-based HPV self-sampling using urine can be used to determine the prevalence of HPV among women living with HIV.

Limitations of the current study include the reliance on self-reported data, which is subject to social desirability bias. To minimise this bias, all research assistants were trained before data collection. Participants who had prior exposure to both methods of sampling of self-sampling and VIA by the nurse may have influenced their responses more towards nurse-led self-sampling. Besides, this study was conducted among women in a rural setting and may not be generalisable to women in urban settings. However, the study provides important information about women’s preference for HPV self-sampling using urine and identifies concerns that may impede the successful implementation of future screening programmes using this method.

In conclusion, this study showed that women living with HIV prefer nurse-led HPV screening to HPV self-sampling using urine. Factors associated with preference for HPV self-sampling using urine are older age (46–65 years), history of sexually transmitted infection, acquisition of CC information from a CHW and acquisition of CC information from television.

Therefore, there is a need for educational programmes to reassure the masses about HPV self-sampling using urine in terms of its accuracy and how it is done. Furthermore, this study showed that CHWs and television may be the most important communication channels to be used for HPV screening and cancer prevention. Human papilloma virus self-sampling needs to be used as a triage test for CC screening and nurse-led CC screening approaches for confirmation of disease among women with HPV-positive results.
